# Whole genome analysis of CRISPR Cas9 sgRNA off-target homologies via an efficient computational algorithm

**DOI:** 10.1186/s12864-017-4225-1

**Published:** 2017-11-17

**Authors:** Hong Zhou, Michael Zhou, Daisy Li, Joseph Manthey, Ekaterina Lioutikova, Hong Wang, Xiao Zeng

**Affiliations:** 1 Department of Mathematical Science, University of Saint Joseph, 1678 Asylum Avenue, West Hartford, CT 06117 USA; 2 Hall High School, 975 N Main Street, West Hartford, CT 06117 USA; 3 Susan L. Cullman Laboratory for Cancer Research, Department of Chemical Biology and Centre for Cancer Prevention Research, Ernest Mario School of Pharmacy, Rutgers, The State University of New Jersey, 164 Frelinghuysen Road, Piscataway, NJ 08854 USA; 4 PBSG, LLC, P. O. Box 771, Braddock Heights, MD 21714 USA

**Keywords:** sgRNA, Off-target homology, Crispr, Cas9, Computational algorithm, Genome wide

## Abstract

**Background:**

The beauty and power of the genome editing mechanism, CRISPR Cas9 endonuclease system, lies in the fact that it is RNA-programmable such that Cas9 can be guided to any genomic loci complementary to a 20-nt RNA, single guide RNA (sgRNA), to cleave double stranded DNA, allowing the introduction of wanted mutations. Unfortunately, it has been reported repeatedly that the sgRNA can also guide Cas9 to off-target sites where the DNA sequence is homologous to sgRNA.

**Results:**

Using human genome and Streptococcus pyogenes Cas9 (SpCas9) as an example, this article mathematically analyzed the probabilities of off-target homologies of sgRNAs and discovered that for large genome size such as human genome, potential off-target homologies are inevitable for sgRNA selection. A highly efficient computationl algorithm was developed for whole genome sgRNA design and off-target homology searches. By means of a dynamically constructed sequence-indexed database and a simplified sequence alignment method, this algorithm achieves very high efficiency while guaranteeing the identification of all existing potential off-target homologies. Via this algorithm, 1,876,775 sgRNAs were designed for the 19,153 human mRNA genes and only two sgRNAs were found to be free of off-target homology.

**Conclusions:**

By means of the novel and efficient sgRNA homology search algorithm introduced in this article, genome wide sgRNA design and off-target analysis were conducted and the results confirmed the mathematical analysis that for a sgRNA sequence, it is almost impossible to escape potential off-target homologies. Future innovations on the CRISPR Cas9 gene editing technology need to focus on how to eliminate the Cas9 off-target activity.

## Background

Derived from the microbial clustered, regularly interspaced, short palindromic repeats (CRISPR) system, the Cas9 endonuclease has become an effective and reliable tool for genome editing in eukaryotes [1–6]. The magnificence of the working mechanism of Cas9 is that it can be guided by a 20-base sgRNA, immediately upstream the short DNA motif of Cas9, the so called protospacer adjacent motif (PAM), to almost any genome loci where the DNA sequence is complementary to the sgRNA [1–4]. The PAM sequence is absolutely required for Cas9 to function and depends on the species of Cas9. For SpCas9, the most used Cas9 species, the PAM sequence is NGG, where N can be either A, C, G, or T. The very first step in making use of the sgRNA-Cas9 system for genome editing is to locate a primary PAM within the target region. Immediately upstream the PAM, the 20 bases of DNA sequence is the guide RNA sequence. Though they can be on either the sense or antisense strand, the PAM and sgRNA sequences must be on the same DNA strand.

Certain rules regarding the design of active sgRNAs have been proposed [6, 7]. As the gene editing mechanism of sgRNA-Cas9 is to generate indels via DNA repairing mechanisms, it is not difficult to understand that for mRNA genes, the target site should better be inside the gene coding sequence and be near the start codon. Another design rule is the GC content. It was found that higher sgRNA GC content could result in higher Cas9 activities [8]. In addition, the design of sgRNA should avoid certain sequences, for example, polyT [7].

One of the most important design rules is to avoid potential Cas9 off-target activity. Unfortunately, a significant number of experiments discovered undesired off-target cleavages by Cas9 at off-target genome sites where the DNA sequences are homologous to the 20-base sgRNA, though with one or more mismatches [7–16]. Considering the large size of some genomes, for example human, mouse and rat genomes, avoiding off-target Cas9 activities immediately becomes the most critical challenge in the application of the sgRNA-Cas9 technology. Systematic research has revealed sequence features governing sgRNA off-target interaction. However, the possible off-target Cas9 cleavages remain a defect and a challenge in sgRNA-Cas9 applications.

The large number of off-target studies of the sgRNA-Cas9 system has led to significant discoveries. Jinek et al. was the first to identify a seed sequence that is less tolerant to mismatches for sgRNA-Cas9 activity [1]. The definition of the seed sequence is generally considered to be the 12 bases on the 3′ end of sgRNA sequence, immediately upstream PAM [1, 10–12]. Mali et al. found that sgRNA-Cas9 system can tolerate one to three target mismatches, and two mismatches inside the seed sequence can eliminate off-target activity [11]. Based on their data, Fu et al. concluded that off-target activity can be observed with up to five mismatches when the concentrations of both sgRNA and Cas9 are relatively high [9]. Hsu et al. discovered that off-target activity depends on the number and positions of the mismatches between sgRNA and target DNA sequence [10]. Lin et al. systematically studied the sgRNA-Cas9 off-target activities when there are indels between target DNA and sgRNA sequences [13]. Their results showed that sgRNAs with low GC content have less tolerance to mismatches. They also found, that a bulge in sgRNA or DNA preserves less Cas9 activity, a result later confirmed by Doench et al. [7].

Making the off-target activity of sgRNA-Cas9 system even more complicated, it has been observed that secondary PAM sequences, in addition to the NGG motifs, can render Cas9 activity [3, 7, 17, 18]. Though these secondary PAMs are far less effective compared to the NGG PAMs, they must be taken into consideration for off-target searches [3, 7]. For SpCas9, the secondary PAMs include NAG, NCG, and NGA [3, 7].

The complexity of the Cas9-sgRNA off-target interaction and the large size of human genome led us to wonder the probability that a given sgRNA sequence has at least one off-target homology. Theoretically, will it be possible to apply the Cas9-sgRNA system without any potential off-target homologies that may introduce unwanted genome editing? In this article, we analyze this question from a mathematical perspective, and then present a very efficient algorithm for sgRNA off-target homology search. This algorithm can complete a whole genome sgRNA design and off-target search in about 40 h under a default setting, an efficiency that cannot be achieved by other available sgRNA software. Via this algorithm, we searched the off-target homologies for all sgRNAs designed for all human mRNA genes. The computational results confirmed our mathematical analysis.

## Methods

The human genome was the sequence source used in this study. As SpCas9 is the most widely used CRISPR-Cas9 system, this study focuses on the mathematical and computational analysis of sgRNA-SpCas9 system. Human mRNA refseq sequence was downloaded from NCBI as the source for sgRNA sequence design. The off-target site search for designed sgRNA sequences were conducted on human chromosome sequences hs_ref_GRCh38.p2 which were also downloaded from NCBI. Computational programs were implemented in Java and executed on a 2016 Dell Precision 7510 laptop computer with Intel(R) Core(TM) i7-6820HQ CPU @ 2.7 GHz and 64.00 GB RAM.

### Mathematical analysis

One crucial assumption made in this mathematical analysis is that the nucleotides A, C, G, T appear randomly at any single location. As there are repeated sequences in human genome, treating the human genome as a purely random combination of A, C, G, T must be regarded as a simplifying assumption. Furthermore, we also assume that human genome has exactly three billion 23-base regions for sgRNA off-target search on one DNA strand. Since the sgRNA can be designed on both the sense and antisense strands, the off-target homologies must be searched on both DNA strands. Thus, the total length of human genome contains six billion 23-base regions. For off-target homology search, we then make the following assumptions:

1. All off-target homologies must have a primary NGG PAM or a secondary PAM immediately downstream the sgRNA binding location.
2. All off-target homologies can have up to four base mismatches within a given sgRNA sequence. If there are at least five base mismatches, the DNA sequence in study is not considered an off-target homology. The reason for defining four instead of five base mismatches as the cut-off is because we have found only one active off-target homology with five base mismatches in the literature, and the off-target activity in that case could be eliminated by lowering both the Cas9 and sgRNA concentrations [9].
3. All off-target homologies can have at most one bulge plus one base mismatch [8, 13]. This implies that a bulge penalty equals three base mismatches.
4. All off-target homologies can have up to two base mismatches or one indel within the seed sequence of sgRNA.
5. No off-target homology can have a DNA bulge that is of two-bases, though an off-target homology can have a RNA bulge of two-bases but with no base mismatch at the same time. No off-target homology can have a bulge of two bases inside the seed sequence.

Based on the above five assumptions, we computed the possible combinations of homologies given a sgRNA sequence. The results are summarized in Table 1. The following explains how the data in Table 1 were obtained.

**Table 1:** Mathematical analysis of the sgRNA off-target homologies

Total combination of 20 bp	1,099,511,627,776
Mismatches in seed sequence	Number of combinations
0	1
1	36
2	594
Mismatches in non-seed sequence	Number of combinations
0	1
1	24
2	252
3	1512
4	5670
Total base mismatches	236,401
DNA bulge with 0 base mismatch	64
RNA bulge with 0 base mismatch	60
DNA bulge with 1 base mismatch	2112
RNA bulge with 1 base mismatch	1968
RNA bulge of two bases	32
Total combinations of homologies	240,637
Off-target homology probability of a 20-base DNA sequence	0.00000021886
Probability of potential PAM	0.2500
Off-target homology probability	0.00000005471

The number of combinations of DNA sequences with different numbers of mismatches is computed by the expression.

$$ \left(\genfrac{}{}{0pt}{}{m}{n}\right)\times {3}^n $$ *,*

where *m* = the length of the DNA sequence in consideration, *n* = number of mismatches. Thus, for the seed sequence of 12 bases, there are 1, 36 and 594 combinations respectively for zero, one and two base mismatches.

As the total base mismatches cannot exceed four, the available base mismatches for the remaining non-seed regions would be zero, one, two, three and four, and can only have a maximum of three or two base mismatches if the seed sequence has one or two base mismatches. So, the total combinations of homologies with up to four base mismatches is computed as:

$$ 1\times \left(1+24+252+1512+5670\right)+36\times \left(1+24+252+1512\right)+594\times \left(1+24+252\right)=236401 $$

The computation of the number of combinations of indels deserves a detailed explanation. There are two cases, DNA bulge, i.e. there is an additional base in the DNA sequence, and RNA bulges, i.e., when there are one or two less bases in the DNA sequence. For both DNA bulge and RNA bulge, there are two sub-cases, i.e. a bulge with zero or one base mismatch. However, for RNA bulge of two bases (there are two bases less inside the aligned DNA sequence), the number of base mismatches must be zero. In addition, if the bulge is inside the seed sequence, then no base mismatch is allowed to be inside the seed sequence.

We start with the DNA bulge with zero mismatches, which means that the 20-base RNA sequence is in fact aligned with a 21-base DNA sequence and all the 20 bases of sgRNA must have an exact match to a base in the DNA sequence. In a 20 vs 20 exact alignment, there are a maximum of 20 positions in the DNA sequence to insert one additional base, and this additional base can be either one of A, C, G, T. There are two additional restrictions when considering a DNA bulge: a DNA bulge can be considered only when there are at least five base mismatches between the sgRNA and DNA sequences (20 bases vs 20 bases) and the introduction of the bulge can trade off more than the number of base mismatches that a bulge penalty equals. Thus, when introducing a bulge inside the DNA sequence, the DNA fragment left of the bulge must be at least four bases such that there are enough base mismatches to be traded off by the bulge. Therefore, there are 16 × 4 = 64 combinations. Via the same logic, the RNA bulge with no base mismatches will have 15 × 4 = 60 combinations.

When there is an indel and a mismatch, the computation becomes a bit more complicated. For DNA bulge, the bulge can be anywhere but the mismatch can only be inside the non-seed region if the bulge is already inside the seed sequence. Thus, the maximum combinations of the indel plus a base mismatch would be

$$ 12\times 4\times \left(\genfrac{}{}{0pt}{}{8}{1}\right)\times 3+4\times 4\times \left(\genfrac{}{}{0pt}{}{20}{1}\right)\times 3=2112 $$

However, for RNA bulge case, the expression would be

$$ 11\times 4\times \left(\genfrac{}{}{0pt}{}{8}{1}\right)\times 3+4\times 4\times \left(\genfrac{}{}{0pt}{}{19}{1}\right)\times 3=1968 $$

The last condition in consideration is the RNA bulge of two bases. Since a two-bases RNA bulge can only be inside the non-seed region, there are only two different ways to form such a RNA bulge because the introduction of such a bulge must trade off at least five base mismatches. The combinations would be

$$ 2\times 4\times 4=32 $$

Based on data in Table 1, the probability for a 23-base single DNA region to be an off-target homology for a given sgRNA sequence is 0.00000005471. Considering the fact that there are six billion 23-base single DNA sequences, The probability for a sgRNA to have no potential off-target homology is 2.67 × 10^−143^, and the expected number of off-target sites is 328.

Based on the above mathematical analysis, it seems that for a given SpCas9 sgRNA sequence, potential off-target homologies in the human genome are unavoidable.

### Computational algorithm

We implemented a sgRNA design and off-target search algorithm in Java. The sgRNA design is based on the rules outlined in [6, 7] with the following exceptions: 1) sgRNA are designed only inside the first half CDS sequence; 2) all sgRNAs do not contain a run of four T or four A.

As the off-target search must be conducted through all the human chromosome sequences, the off-target search of sgRNA can be very time expensive. The high efficiency of our off-target search process comes from two critical algorithmic innovations which are explained below in detail.

The first innovation is that an indexed database based on the seed sequence variations is dynamically constructed before any homology search work starts. Based on assumption 4, for a DNA region to be an off-target homology of a given sgRNA, it must have a good alignment with the sgRNA seed sequence such that there should be at most two mismatches or one indel. Hence, the off-target homology search starts with finding those DNA sequences that are variations of the sgRNA seed sequence. The seed sequence consists of 12 bases, so there are 4^12^ different 12-base variations in total. If we assign 0, 1, 2, 3 to A, C, G, T respectively and convert DNA sequence to a base-4 number system, then each 12-base variation can then be represented as a unique integer using the expression $\sum_{i=0}^{11}N\times {4}^i$, where *N* = 0, 1, 2, 3, representing A, C, G, T respectively.

Since the package was implemented in Java whose *int* data type can only hold integers ranging from −2^31^ to 2^31^-1 and the human genome has about three billion base pairs, i.e. six billion bases, we decided to divide the 24 chromosomes into two groups with roughly equal number of nucleotides. For each group, a two-dimensional array G_ij_ is constructed as follows: *i* = the integer value of each 12-base sequence, the row G[i] stores all the positions of the 12-base sequence (equivalent to integer *i*) in the group of chromosomes. A positive G[i][j] indicates that the position is on the sense strand while a negative G[i][j] means that the 12-base sequence is found on the anti-sense strand. Given the integer G[i][j], a conversion system matches it to a specific chromosome, a specific NT record, and a specific position inside the NT sequence. An important tip in constructing the two-dimensional array G_ij_ is that G_ij_ only stores the location information of those 12-base sequences followed by a primary PAM or a secondary PAM.

Given a 20-base sgRNA sequence, based on its 12-base seed sequence, all variations of its 12-base seed sequence are generated according to Assumption 4, which are interpreted as: 1) a variation can have at most two mismatches with this seed sequence; 2) a variation can have at most one indel when aligned against the seed sequence. The homology search algorithm then finds all the exact positions inside each NT record for all the different variations very quickly and then uses a dynamic programming algorithm to determine if there is an off-target homology at each position.

The second innovation is the efficient dynamic programming algorithm for homology determination. The dynamic programming algorithm is illustrated in Table 2.

**Table 2:**
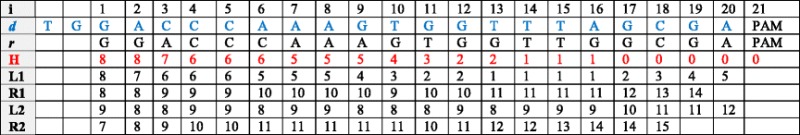
Dynamic programming illustration. i: the subscript of the table; *d*: the DNA sequence; *r*: the sgRNA sequence; H: the number of base mismatches; L1: 1-base DNA bulge; R1: 1-base RNA bulge; L2: 2-base DNA bulge; R2: 2-base RNA bulge

The construction of Table 2 is explained as follows. Given a DNA sequence marked as ***d*** and a sgRNA sequence marked as ***r***, for ***d*** to be an off-target homology of ***r***, it must have a PAM (either primary PAM or secondary PAM) that aligns with the PAM of ***r***.
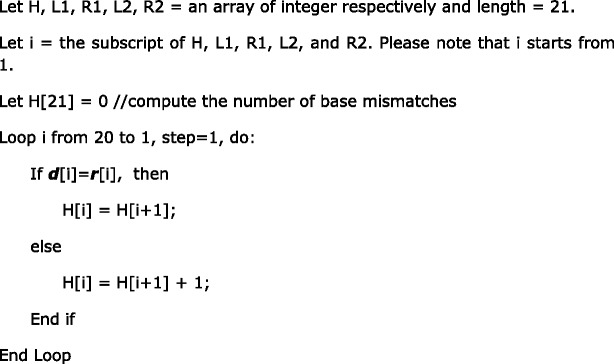


For DNA bulges of 1 base or 2 bases, which are marked as L1 and L2 respectively in Table 2, the values are computed as:
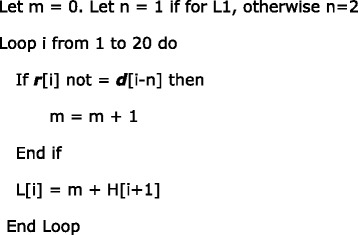


For RNA bulges of 1 base or 2 bases, which are marked as R1 and R2 respectively in Table 2, the values are computed as:
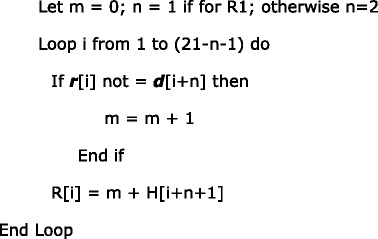


The above algorithm computes the number of base mismatches only, which are the values in Table 2. For L1, L2, R1 and R2, as there is a specific bulge for each case, the total number of mismatches should add the specific bulge penalty. In our default setting, a bulge penalty equals three base mismatches (counted as two if inside the seed sequence), a RNA bulge extension penalty equals one base mismatch, and a DNA bulge extension penalty equals two base mismatches. Thus in Table 2, when L1 is computed, though it is shown that L[13] = L[14] = L[15] = L[16] = 1, they are in fact = 1 + DNA bulge penalty = 4. The result shows that by shifting the 5′ fragment (up to either the 13th, 14th, 15th, or 16th base) one base to the left, we can achieve an alignment with only one base mismatch and one DNA bulge.

The above algorithm illustrates the general condition. There are some special cases that the implementation must also consider:

- Since the seed sequence has more stringent requirements on the number of mismatches, the number of base mismatches and indels within the seed sequence should be counted and stored to determine whether or not a specific alignment should be considered as an off-target homology. In the example shown in Table 2, though the case of L1 can achieve a good alignment with only one base mismatch and one DNA bulge, ***d*** is eventually not considered a homology to ***r*** because both the DNA bulge and the base mismatch are inside the seed sequence,
- There are a total of five cases that are computed in this algorithm: H, L1, R1, L2, R2. If in one case ***d*** is found to be a homology to ***r***, there is no need to go on to the next case.
- For cases L1, R1, L2, and R2, a shortcut can be applied. If (m + bulge penalty) become larger than the number of base mismatches allowed, there is no need to continue computing for that case because it is guaranteed that the alignment represented by this case is not a homology.

## Results and discussion

We first simulated a human genome of size three billion base pairs in which A, C, G, T are randomly distributed. With this simulated genome, we examined the off-target homologies for 1,000,000 sgRNAs randomly designed from the simulated genome and the 1,876,775 sgRNAs designed for the 19,153 human mRNA genes based on the above design rules. The off-target homology search identified 326 homologies per sgRNA in average for the group of 1,000,000 sgRNAs and 325 homologies per sgRNA in average for the group of 1,876,775 sgRNAs. Both results are fairly close to the mathematically expected 328 homologies. In fact, the mathematically expected values should be slightly larger than the computational experimental values because of two reasons. The first reason is that the mathematically calculated number of combinations for the case with one indel plus one base mismatch is the possible maximum number. The real number should be slightly smaller. The second reason can be explained by using the sequence alignment (DNA) ACCCCT/acccct (RNA) as an example. Removing any C will generate the same RNA bulge ACCCT/acccct, i.e. the computational experiment will detect one RNA bulge while the mathematical model would count four times. Overall, in agreement with our mathematical model, no sgRNA was found to be free of homologies with the simulated genome.

The computational experiment with human genome identified that only two out of the 1,876,775 sgRNAs were validated to be free of off-target homology. This confirms our mathematical analysis that theoretically, it is almost impossible for a sgRNA to have no potential off-target homologies. A total of 1,415,606,013 off-target homologies were found, indicating 754 off-target homologies per sgRNA. This number is significantly larger than the mathematical expected value. We believe that the large discrepancy was resulted from the fact that human DNA sequence is not a random composition of A, C, G, T. There are a large number of repeated sequences in human genome [19]. As we once pointed out [20], some sgRNAs with repeated sequences have an unusually large number of off-target homologies, which contributes to the large discrepancy.

It is worth to point out that of the 1,415,606,013 homologies, about 2.70% are with indels. Thus, even though the off-target homologies are mostly base mismatches, indels are a significant portion of off-target homologies and should be considered. Some sgRNA off-target search algorithms, for example, CasFinder and CRISPOR, do not detect indels, and thus miss a significant number of off-target homologies [21, 22].

The time cost to complete a whole genome sgRNA design and off-target homology examination is mostly on the homology examination. The time cost is a linear function of the number of sgRNAs. Furthermore, based on our homology examination algorithm, it is easy to understand that the time cost is also a function of the off-target homology definition. Under our default homology examination settings, the time cost to complete the whole genome design and off-target examination for the 1,876,775 sgNRAs is about 40 h. It is roughly about 77 s for every 1000 sgRNAs.

Compared with CasFinder which is built upon Bowtie, our package is much more efficient. Under a similar homology examination setting (the seed sequences allows maximum two mismatches, the 20-base sequence allows totally up to four base mismatches but no bulge, and the secondary PAM is only NAG), CasFinder took 624 h to complete the design and off-target examination of its 927,104 sgRNAs while our algorithm took about 22 h to examining 1,876,775 sgRNAs [21]. Roughly speaking, our algorithm is about 57 times faster than CasFinder.

Cas-OFFinder employed a similar strategy as our algorithm except that they first computed the variations of the 20-base guide sequence with up to certain number of mismatches [23]. With each varied sequence, they tended to find an exact match in the genome. We also compared our algorithm’s efficiency with theirs under the same conditions: up to five base mismatches, no indels, and only consider the NGG and NAG PAM. Cas-Offinder’s maximum speed via GPU is about 3.01 s per sgRNA sequence. However, when comparing the CPU efficiency, Cas-Offinder’s maximum speed is about 60.03 s per sgRNA sequence, while ours is about 3.15 s per sgRNA sequence.

Because each sgRNA has very high probability to have off-target homologies that can result in off-target Cas9 activity, avoiding potential off-target activity is in fact the most challenging and critical factor in designing sgRNAs. In addition to its efficiency, another advantage of our algorithm is that it guarantees to find all the potential off-target homologies based on the off-target homology setting. It has been reported that a few tools are likely to miss significant number of potential homologies [22, 24]. Thus, we compared our algorithm with CRISPOR (http://crispor.tefor.net/) and Cas-OFFinder (http://www.rgenome.net/cas-offinder) which were considered to be superior in locating off-target homologies [22]. Using the EMX1 guide sequence (GAGTCCGAGCAGAAGAAGAA) as an example, Table 3 shows that our algorithm achieves as good as both Cas-OFFinder and CRISPOR.

**Table 3:** Comparison between CRISPOR, Cas-OFFinder and the proposed algorithm on off-target homology search for EMX1 sgRNA guide sequence

	Number of off-target homologies identified				
Number of base mismatches	0	1	2	3	4
CRISPOR	0	0	6	38	296
Cas-OFFinder	0	0	1	18	273
Our Algorithm (with Secondary PAM)	0	0	6	87	1227
Our Algorithm (without secondary PAM)	0	0	1	18	273

Under exactly the same conditions, our algorithm found exactly the same off-target homologies as Cas-OFFinder and CRISPOR did. The only difference is that, by default, our algorithm searched for off-target homologies anchored with all the secondary PAMs including NAG, NCG and NGA. The web-tool of Cas-OFFinder did not search for any secondary PAM, while CRISPOR considered only a few PAMs (NAG, AGA, GGA, TGA).

The large expected number of homologies for each sgRNA has been motivating scientists to search for different solutions. A double nicking approach was then introduced to enhance genome editing specificity [11, 25]. The double nicking method is based on the Cas9 nickase mutant that can only break one single strand of DNA. To obtain a double stranded cleavage, simultaneous nicking via two individual sgRNAs each targeting a different strand is necessary [25]. The offset, the distance between the 5′ ends of the two sgRNA sequences (sgRNA pair), must be between −4 and 20 for the paired nicking to work well, and if the offset of the paired sgRNAs is less than −34 or larger than 110 bases, the paired-sgRNA-Cas9 system completely loses its efficacy [25]. Thus, a potential off-target homology for paired sgRNA nicking must have two single off-target homologies positioned in a way that their offset is between −34 and 110 bases inclusive. After 387,679 sgRNA pairs were designed for the 19,153 mRNA genes, 175,712 sgRNA pairs were found to be free of off-target homologies, covering 14,665 mRNA genes. This confirms that the double nicking method is much more reliable than the original SpCas9-sgRNA system in avoiding off-target homologies, a finding reported before [16, 25].

## Conclusions

A novel and efficient sgRNA homology search algorithm was introduced in this article. Via this algorithm, genome wide sgRNA design and off-target analysis were conducted and the results confirmed the mathematical analysis that for a sgRNA sequence, it is almost impossible to escape potential off-target homologies. Future innovations on the CRISPR Cas9 gene editing technology need to focus on how to eliminate the Cas9 off-target activity.
